# The revival of spiritual practices: factors influencing the “seeking deities and offering prayers” behavior of China’s Generation Z on social media in an atheistic context

**DOI:** 10.3389/fpsyg.2024.1485265

**Published:** 2025-01-30

**Authors:** Jing Wang, Balamuralithara Balakrishnan, Xiaohui Wan, Qirui Yu, Qiqi Ye

**Affiliations:** ^1^School of Culture and Communication, Putian University, Putian, China; ^2^Faculty of Art, Sustainability and Creative Industry, Sultan Idris Education University, Tanjong Malim, Perak, Malaysia; ^3^Faculty of Human Development, Sultan Idris Education University, Tanjong Malim, Perak, Malaysia; ^4^Faculty of Creative Arts, University Malaya, Lembah Pantai, Kuala Lumpur, Malaysia; ^5^School of Literature and Communication, Wenzhou University of Technology, Wenzhou, China

**Keywords:** digital religious behavior, Chinese Generation Z, social media, theory of planned behavior, social identity, empathetic willingness

## Abstract

**Introduction:**

Over the past decade, there has been a growing focus on the study of how religion and technology intersect, particularly within the field of digital religion studies. In recent years, digital religious activities have emerged on Chinese social media platforms, with the sharing and promotion of content related to religious activities becoming increasingly popular. In a country like China, which adheres to atheistic ideology, the emergence of religious activities among the Generation Z youth provides a unique case for academic research. However, there is very limited research on digital religious behavior in mainland China. This study fills this gap by extending the Theory of Planned Behavior (TPB) to predict additional influencing factors of digital religious intentions and behavior.

**Methods:**

This study employed a quantitative design, disseminating surveys via Sina Weibo and the Douyin platform. We collected a total of 525 valid responses. This study aims to deeply explore the social and psychological factors generated by digital religious activities on social media platforms, particularly how they stimulate Chinese Generation Z youth to participate in digital religious activities.

**Results:**

The results show that attitudes toward digital religion, perceived behavioral control, social identity, and empathetic willingness are predictors of intentions, while social norms are not. Intentions significantly predict users’behavior on social media platforms such as Weibo and Douyin. Moreover, empathetic willingness and social identity fully mediate the effects of subjective norms, perceived behavioral control, and attitudes on intentions. Consequently, these behaviors generate impact, indicating the presence of multiple mediation effects.

**Discussion:**

Unlike the societal context of theistic nations, there is a pronounced atheistic inclination within Chinese society. Consequently, subjective norms do not influence the digital religious behaviors of Chinese youth. Among contemporary Chinese youth, participation in digital religious practices is a temporary, secular activity undertaken only as a comforting behavior when anxiety becomes unmanageable or surpasses their threshold of tolerance. In contemporary China, young people face immense pressures stemming from workplace competition, social interactions, and economic burdens. Social media offers these young individuals broader opportunities for connection, community formation, and identity construction, as well as various possibilities for organizing their social lives. Consequently, on one hand, they turn to online religious avenues, seeking understanding from others through shared experiences, thereby obtaining emotional solace and comfort. On the other hand, they look to spiritual beliefs to alleviate anxiety, resolve confusion, and gain psychological comfort through emotional exchanges. Therefore, digital religion can, to some extent, be viewed as a form of social-technological empowerment, providing contemporary youth with a new “pressure valve” to facilitate emotional relief, comfort, and tension alleviation.

## 1 Introduction

Over the past decade, there has been a growing focus on the study of how religion and technology intersect, particularly within the field of digital religion studies (Campbell and Bellar, 2022). New communication technologies have been found to facilitate religious purposes (Al-Zaman, 2020; Udupa, 2017; Campbell, 2024) and increase opportunities for integration into religious communities (Campbell and Connelly, 2020; Udupa and McDowell, 2017; Lundby and Evolvi, 2021). Current studies on digital religion increasingly focus on how different groups negotiate their interpersonal and spiritual relationships across multiple realms of their online and offline lives (Kergel et al., 2020; Campbell, 2017; Aguilar et al., 2017; Lam and Mansouri, 2021).

In recent years, digital religious activities have emerged on Chinese social media platforms, with the sharing and promotion of content related to religious activities becoming increasingly popular (He et al., 2024; Gao et al., 2024). These digital religious contents and activities significantly influence the behavior and attitudes of young users (Zhang, 2023; Statista, 2020). For instance, some young people seek academic progress by burning incense and praying online, while others wish for career advancements or salary increases by sharing religious videos to seek good luck. Phrases like “young people choose to offer incense between working and advancing oneself” frequently appear on Chinese social media platforms. Some scholars term this phenomenon a “seeking deities and offering prayers” trend (Shen, 2020). Moreover, offline data show that in February 2024, temple scenic spot orders in mainland China increased by 310% year-on-year, with nearly 50% of the ticket bookers being young people aged 18–25. This generation, generally born between 2000 and 2012 (Dimock, 2019), is known as Generation Z. The primary motivations for China’s Generation Z to engage in “seeking deities and offering prayers” include wishing for a successful career, academic achievements, financial luck, and romantic relationships.

However, an equally evident fact is that China has the largest population of non-religious individuals in the world. According to the Chinese Family Panel Studies (CFPS, 2012), only 10% of Chinese people identify as religious. In contrast, globally, approximately 59% of the population identifies with a religion (Pew Research Center). Additionally, China is governed by the Chinese Communist Party (CPC), which upholds atheistic principles (Yang and Huang, 2018). The CPC, the second-largest political party in the world, had 99.185 million members as of December 2023, all of whom are prohibited from holding religious beliefs (People’s Daily, 2016). Non-CPC members are also exposed to ideological education starting from their primary and secondary school years (Xie et al., 2017). Historically, while China boasts a rich religious heritage, the state has maintained a restrictive and cautious stance toward religion. In the late 1950s, the Chinese government regarded religion as an exploitative system that should be abolished (Wei, 2024). This led to widespread destruction of religious sites, cessation of religious activities, and the dissolution of clergy (Ashiwa and Wank, 2009). Although in 1976, the government re-recognized the legitimacy of religion, reestablished religious institutions, and began rebuilding religious sites, stricter regulations were subsequently imposed (Ashiwa and Wank, 2009; Leung, 2018). On one hand, the Chinese government officially claims to protect freedom of religious belief, but it explicitly excludes folk religions, superstitions, and cults from this protection (Overmyer, 2003, pp. 385–410). On the other hand, the state has consistently promoted atheism (Overmyer, 2003, p. 411). As Potter (2003) noted, a hallmark of China’s cautious attitude toward religion is its emphasis on educating younger generations in historical materialism and atheism. Consequently, despite constitutional guarantees of religious freedom (Lin, 2015), China remains the country with the lowest percentage of religious believers (Yang and Huang, 2018). It is evident that China, as a country adhering to atheistic ideology, has an official stance on religion characterized by restrictions and prudence. Despite these policy and socio-historical limitations, the ritual of “seeking divine intervention” in Chinese folklore is widespread among both religious and non-religious individuals, particularly among contemporary young Chinese who increasingly engage in religious and spiritual activities on social media platforms. Therefore, the unique trajectory of religious development in China provides an extraordinary context for academic research.

Furthermore, Chinese Generation Z seems to face more severe pressures than their peers in other regions, encompassing employment, interpersonal relationships, and marriage (Fu, 2018; Wu et al., 2024). The politically polarized climate, complex digital ecosystem, and dramatic changes in work and daily life (Stahl and Literat, 2023; Laufer et al., 2010) collectively create distinct impacts on contemporary youth compared to previous generations (Parker and Igielnik, 2020; Stahl and Literat, 2023). These pressures in modern society drive young people towards religion (Sangren, 2012; Wang, 2021; Xia, 2023). Specifically, in the Chinese context, the desire to control one’s “fate” is a significant factor for the public’s participation in religious rituals, particularly when making crucial decisions and hoping that these wish-making ceremonies bring them good fortune (Shen, 2023). Xia (2023) interprets the current trend of “seeking deities and offering prayers” among the youth as a component of the subculture of Generation Z. She posits that such behavior implicitly signifies a failure of social identity among young individuals amidst intense societal transformations. It denotes a method of self-therapy sought after deviations emerge between self-fashioning and the influence exerted by others.

These behaviors transcend traditional religious frameworks, reflecting deeper characteristics of contemporary Chinese social structures and the psychological needs of young groups. Research shows a close relationship between the dissemination of religious beliefs and media technology (Yamane, 2016; Campbell, 2017). The unique aspects of social media’s interaction modes, sharing settings, and content production methods are considered key drivers of this change (Jiang et al., 2016; Lajnef, 2023). In a country like China, which adheres to atheistic ideology, the emergence of religious activities among the youth provides a unique case for academic research. The internet, being the “home ground” for Generation Z (Stahl and Literat, 2023; Wallaroo Media, 2021) and a crucial space for their online collective expression (Literat and Kligler-Vilenchik, 2021; Delgado et al., 2023), serves as a valuable window to understand young people’s attitudes and views. Furthermore, the resurgence of spiritual participation among a traditionally secular demographic raises intriguing questions about the underlying motivations for these behaviors and the role of social media in shaping these religious inclinations.

Existing literature documents the suppression and regulation of religious activities in China’s history, along with the government’s efforts to promote secularism (Yang, 1961; Potter, 2003). However, traditional beliefs and customs persist and, in some cases, have experienced a revival (Feuchtwang, 2003; Overmyer, 2009; Sangren, 2012; Chan, 2014; Chau, 2008; Lin, 2020; Shen, 2023). The role of social media in facilitating this revival has been acknowledged and recognized (Abbasi et al., 2023; Wilkins-Laflamme, 2022) but has not been widely explored. Moreover, within the context of Western research, it has been corroborated that there exists a positive correlation between possessing faith and experiencing happiness (e.g., Diener and Seligman, 2002; Ferriss, 2002; Lim and Putnam, 2010; Stark and Maier, 2008). However, this hypothesis has seldom been empirically tested in contemporary China (Lu and Gao, 2017). Current studies on the impact of social media on Chinese society primarily focus on political expression (Fung et al., 2023; Chen et al., 2021), community building (Craig et al., 2021), and cultural consumption (Wilska et al., 2023; Kamboj and Sharma, 2023). Research on religion in the Chinese context has primarily focused on the revival and development of religion across different regions (Goossaert and Plarmer, 2011; Laliberté, 2011; Madsen, 2011; Overmyer, 2003), religious policies (Yang, 2011), and regional folk religions (Dean, 2003; Lipman, 2011).

Media, religion, and culture studies have become a rapidly growing interdisciplinary field (Campbell, 2017), with several classical theories used to explain the interaction among these elements. The Theory of Planned Behavior (TPB, Ajzen, 1991) is one of the most influential and effective social psychology theories for predicting human behavior in various contexts (Jiang et al., 2016). TPB has also demonstrated significant explanatory power and influence in understanding and predicting digital religious behaviors in online activities (Campbell, 2017; Lundby, 2013; Ang et al., 2023). This theory posits that individual behavior is predominantly influenced by behavioral intentions, which are shaped by three key motivating factors: attitudes, subjective standards, and perceived behavioral control (Jiang et al., 2016). Previous studies applying TPB have shown that the predictive power of the model improves when additional TPB variables are introduced. However, a few studies have incorporated adolescents’ needs for social identity and emotional fulfillment when identifying TPB components (Khalil et al., 2023). Nevertheless, there is a scarcity of theory-driven research on digital religious behavior in China. Most of the studies found in the literature review were conducted in countries like the United States and Australia (Juan et al., 2022; Chen and Huang, 2019; Marwantika, 2022; Acevedo and Chaudhary, 2015; Hadad and Schachter, 2011; Lam and Mansouri, 2021), and few studies have been conducted in the context of mainland China (Overmyer, 1990; Leamaster and Hu, 2014). Consequently, while TPB demonstrates strong explanatory power for digital religious behavior in countries with established religious beliefs, its applicability in China remains uncertain.

This study aims to deeply explore the social and psychological factors generated by digital religious activities on social media platforms, particularly how they stimulate Chinese youth to participate in these activities. The study adopts an expanded version of the TPB as the theoretical framework, which includes traditional factors such as attitude, subjective norms, and perceived behavioral control, and also explores two additional factors: social identity and empathetic willingness. The phenomenon of “seeking deities and offering prayers” among contemporary Chinese youth essentially reflects their underlying psychological states of anxiety, confusion, and inner conflict (Xia, 2023). In China, external factors, including economic downturns and employment difficulties, have increasingly deprived young people of social support and recognition (Zhang, 2023). At the same time, atomized individuals often find themselves isolated due to the lack of assistance from social and public institutions (Wu and Sun, 2024). Media has become a key sociocultural mechanism for modern group identity formation (Leung and Lee, 2015), and young people rely on social media to gain social recognition as a way to resist societal alienation (Wu and Sun, 2024). Therefore, this study incorporates social identity into the model. Additionally, endogenous factors include the need for intimate emotional connections (Zhang, 2023). “Empathetic willingness” offers a cross-sectional view of psychological and emotional requirements. Empathy is a process that includes two distinct components: emotional sharing and cognitive regulation, and it serves as a mechanism that connects individual emotional experiences with a universal link to well-being (Batson et al., 1997). The current situation in China, where youth commonly face excessive psychological stress and their negative emotions have been long neglected (Xia, 2023), necessitates the use of empathy to express resistance to these negative feelings (Chen and Ma, 2023). Therefore, this study suggests that empathetic willingness acts as a mediating factor, prompting Chinese youth to use solemn and respectful religious rituals on social media as emotional bonds and a desire to create emotional connections with others. However, the role of empathy in communication has been relatively understudied in past research (Chen and Ma, 2023). Thus, this study introduces the need for empathetic willingness in the model to explain the digital religious behavior of Chinese youth. These factors help provide a comprehensive perspective to understand the religious behaviors of Chinese youth under the influence of self-identity construction on social media. This multidisciplinary research method helps show how the religious beliefs and actions of younger people have changed over time in a rapidly changing and complex society. It does this by looking at communication studies, sociology, and psychology to find out more about modern Chinese society and the mental needs of young people. The ultimate objective of this research is not only to provide new insights in the academic field but also to provide guidance for social policy, education, and communication practices. Understanding the importance and mechanisms of religious belief in young people’s lives, as well as how social media influences these young people’s faith intentions, can contribute to a more comprehensive understanding of social and cultural issues.

This study explores two primary objectives. Firstly, we will examine the psychological and behavioral mechanisms underlying the online religious behavior of Chinese young people in an atheist country. Secondly, we will examine the influence of social media on Chinese youth’s digital religious beliefs and behaviors using the extended TPB. To improve the TPB framework, we should consider adding two additional factors, namely social identity and empathetic willingness, in order to gain a deeper understanding of this complex phenomenon and thus provide relevant social policies and media practices with new insights and explanatory power.

## 2 Theoretical background for prediction and explanation

### 2.1 The original theory of planned behavior model

In explaining human intentions and behaviors, the TPB has become one of the most important theories (Armitage and Conner, 2001). TPB suggests that attitudes, normative beliefs, and control beliefs independently determine the willingness to take (or not take) action on certain behaviors, and that willingness is the direct cause of behavior (Ajzen, 1985, 1991; Fishbein and Ajzen, 1975). Many scholars have adopted the TPB theory to predict and explain online behaviors, such as selfie behavior on social media (Baker and White, 2010; Chen and Huang, 2019) and tourism participation behaviors on social networking sites (Müller-Pérez et al., 2023; Prieur et al., 2023; Siddiqui and Hamid, 2023). These studies confirm that attitudes, subjective norms, and perceived behavioral control have a positive impact on these behaviors. In terms of predicting religious behaviors using the TPB theory, the study by Shah Alam and Mohamed Sayuti (2011) confirmed that attitudes, subjective norms, and perceived behavioral control have a significant positive impact on the intention to purchase halal food (Chok, 2013). Similarly, the study by Kasri and Ramli (2019) confirmed that subjective norms and perceived behavioral control positively correlate with Indonesian Muslims’ donation behaviors to mosques.

According to Ajzen (1985), one of the main advantages of the TPB is its ability to incorporate additional variables to enhance its explanatory power. Some researchers believe that including social identity (Hagger and Chatzisarantis, 2006; Jiang et al., 2016) and empathetic willingness (Dovidio et al., 2010; Luchsinger, 2020; Zhao et al., 2013) significantly increases the model’s explanatory power. However, no research has been found that includes both of these factors in the TPB model, nor has there been much exploration of these variables’ explanatory power in social network contexts. Therefore, this study uses an extended TPB model to explain the intentions and behaviors generated by digital religious activities on social media platforms, including the basic TPB model as well as social identity and empathetic willingness.

### 2.2 Digital religious behavior

Digital religion is defined as the technological and cultural space created when discussing how religion is integrated or fused and its potential impact on religious expression and beliefs (Lövheim and Campbell, 2017; Campbell, 2017; Campbell and Evolvi, 2020). Religious behavior is considered one of the socio-cultural value systems related to moral behavior (Singhapakdi et al., 2013; Zarkada-Fraser and Fraser, 2002; Singhapakdi et al., 2013; Jiang et al., 2016). Over the past decade, research on religious communication and technology has been discussed more frequently under the umbrella of digital religion studies (Campbell, 2020; Campbell and Connelly, 2020). Digital religion research focuses on closely examining how digital religion influences the characteristics of online culture, such as its interactivity and convergence (Cheong et al., 2012; Campbell, 2017). Current research on digital religion increasingly focuses on areas such as how religious actors negotiate their relationships and spiritual activities between online and offline life domains (Cheruvallil-Contractor and Shakkour, 2015; Neriya-Ben Shahar, 2017), how personal spirituality and religious beliefs are shaped in digitally-driven communication practices (Sumiala and Korpiola, 2017; Lundby, 2013), and individuals’ daily spiritual patterns (Bellar, 2017; Campbell, 2017).

However, as Lagerkvist (2017) pointed out, other key humanistic areas often overlooked in digital religion studies, such as gender and age, influence the religious and digital contexts (Lövheim, 2013). Therefore, Campbell (2017) suggested that studies on classic themes in digital spaces and religious practices, such as specific groups and their faith activities, should be encouraged (Aguilar et al., 2017). It has been found (McClure, 2016; Campbell, 2017; Coman and Coman, 2017; Campbell, 2020; Dawson and Cowan, 2004; Karaflogka, 2007; Possamai and Turner, 2012; Wagner, 2012) that young people who use social media are more likely to believe it is feasible for them to choose their religious beliefs and practices from a variety of religious traditions, a perspective developed by their interaction with other online users.

### 2.3 Digital religious intention

Digital religious intention refers to the subjective probability judgment of individuals’ tendencies toward or handling of objective matters (Alhouti et al., 2015). Intentions are considered proximal determinants of behavior, with positive intentions influencing actual user behavior (Dhir et al., 2019). Previous studies have posited that religious intentions include “main utilitarian motivations underlying religious behavior” (Atakan et al., 2008; Kashif et al., 2017), “religious participation for selfish reasons” (Davidson and Farquhar, 2014), and “motivations based on the intrinsic goals of religious traditions themselves” (Syahirah et al., 2017), as well as “commitment to and participation in more intrinsic spiritual goals” (Lim, 2016; Hassan et al., 2024). Research on the association between intentions and actual behavior in social network contexts has confirmed the predictive power of intentions (Campbell and Evolvi, 2020; Shah Alam and Mohamed Sayuti, 2011; Lichy et al., 2023; Kasri and Ramli, 2019). Dale et al. (2020) pointed out that in examining the intention to continue participating on Facebook, there is a strong correlation between intention and actual behavior. This has also been proven in the context of teenagers’ social media use (Bateman and Valentine, 2010), where stronger intentions lead to more related behaviors, especially in terms of continuous usage behavior (Bhutto et al., 2023). Many studies related to virtual temples and churches have shown that individuals’ religious customs and intentions in digital religion influence the elements involved in specific rituals (Campbell and Vitullo, 2016; Chen et al., 2020; Koufie et al., 2024) and also affect new forms of participation and prayer practices (Morehouse and Austin, 2022).

### 2.4 Subjective norms

Subjective norms are defined as the influence of significant others (such as friends, family, and peers) on users’ behavior (Ajzen, 1991). It has been found that people are more likely to engage in a behavior if they believe that certain significant others consider such behavior appropriate, and these individuals are typically their friends, family members, and other relatives (Alsuhaymi, 2018; Hua and Pang, 2024). Conversely, if significant others do not encourage a behavior, people are less likely to perform it (Rhodes et al., 2020). Subjective norms are significantly related to users’ behavior in using Internet services and have been consistently confirmed (Lonkila and Jokivuori, 2023; Zhong and Wu, 2023), including daily digital consumption behavior (Dale et al., 2020; Hameed et al., 2019), broadband usage and adoption (Dwivedi, 2005; Lu et al., 2009), continued intention to use mobile data services (Kim et al., 2016), and users’ behavioral intentions in online spaces (Marston, 2024). Significant positive effects have been found on SNS continuous use intention (Dhir et al., 2019; Dixit and Prakash, 2018; Jo, 2022). The respondents in this study are 18- to 25-year-old Chinese youth. In Chinese culture, collectivism is highly emphasized, and the one-child policy has been implemented for decades (Wu et al., 2021; Tsui and Ngo, 2016; Juan et al., 2022). The participants in our study were born between 2000 and 2006, a period in China characterized by a dominant united and collectivist culture (Wu et al., 2021). It is common practice for individuals to conform to collective norms (Xia, 2023). The perception of digital religion by peers or other group members strongly influences the respondents’ intentions (Shen, 2023).

### 2.5 Attitudes towards digital religion

Attitudes are defined as an individual’s evaluation or appraisal of a behavior (Takahashi et al., 2021; Ajzen, 1991, 2020). Religiosity refers to the strength of belief concerning the existence of God (Hari Adi and Adawiyah, 2018), the relationship between humanity and divinity, adherence to religious principles, and compliance with religious practices in various aspects of life (Campbell and Evolvi, 2020). Individuals who strictly adhere to religious principles tend to score high in religiosity and are more likely to exhibit attitudes that promote moral intentions (Weaver and Agle, 2002; Ishak et al., 2023). Therefore, digital religious attitudes can explain individuals’ responses to digital religious behaviors, such as preferences and dislikes, as well as emotional feedback, such as whether an experience was pleasant. Previous research has found that attitude is a significant predictor of religious intentions and behaviors (Kashif et al., 2017; Hameed et al., 2019; Suleman et al., 2021). Therefore, a positive attitude is expected to significantly increase the likelihood of engaging in digital religious behavior. Scholars have discovered that in the context of technology use, attitudes influence users’ intentions to continue using the technology (Campbell and Evolvi, 2020; Ali et al., 2020). Similarly, in the context of social networking sites (SNS), the vast majority of research has found that attitudes significantly impact SNS users’ religious behavioral intentions (Al-Ghaith, 2016; Dhir et al., 2019; Morehouse and Austin, 2022; Collins-Kreiner, 2020). Therefore, we hypothesize that the attitudes of young social media users play an important role in influencing their ongoing intentions toward digital religion.

### 2.6 Perceived behavioral control

Perceived behavioral control refers to the extent to which people perceive the ease or difficulty of performing a digital religious behavior based on past experiences and anticipated barriers. It is an important predictor of intentions (Ajzen, 1991; Paul et al., 2016). In other words, it is not entirely under personal voluntary control (Collins-Kreiner, 2020). Digital religious behavior among Chinese youth may be constrained by devout environmental factors, religious policies (Shen, 2023), or other recognized behavioral norms or rules (Muthu, 2019). Recent studies emphasize expanding the structural scope of moral decision-making research by considering the factors influencing individual moral intentions and behaviors, both internally and socially constructed (Kashif et al., 2017). PBC is an individual characteristic, while moral norms are internal standards (Kashif et al., 2017; Masri Bin et al., 2023). Existing research has shown strong support for PBC’s influence on intentions and behaviors in moral decision-related studies (Chen and Tung, 2014; Yadav and Pathak, 2016; Lin, 2020). Moreover, some studies have demonstrated that once a sense of control is instilled, attitudes begin to act as a positive stimulus triggering moral decisions (Kashif et al., 2017; Goles et al., 2008). Two major factors that may affect PBC include the availability of necessary resources and opportunities to perform the behavior (Jiang et al., 2016; Duarte et al., 2024). When individuals become aware that they can obtain the necessary resources and opportunities to effectively carry out a behavior, they typically exhibit a high level of PBC (Hauptmann and Gerlach, 2010; Jiang et al., 2016). This, in turn, results in a stronger intention to engage in the action. Prior studies have established that PBC has a crucial role in predicting intentions within virtual communities (Lin, 2006) and the adoption of instant messaging (Jiang et al., 2016; Lu et al., 2009). Religious beliefs shape people’s identity, thereby increasing their perceived behavioral control (Kashif et al., 2017). Therefore, we expect PBC to positively predict Chinese youth’s digital religious intentions (DRI) toward digital religion.

### 2.7 Additional variables: social identity and empathetic willingness

#### 2.7.1 Social identity as a mediator

Social Identity Theory posits that social identity arises from an individual’s perception of their belongingness to one or several social groups (Ellemers et al., 1999; Jiang et al., 2016), together with the emotional importance attached to that belongingness (Jiang et al., 2016; Fielding et al., 2008; Tajfel, 1974). Individuals within the same group are more likely to act according to the behaviors of their in-group members and differ from those outside the group (Makhubela, 2020). Research by Jiang et al. (2016) indicates that in social media, individuals who perceive support from members of their group may show a greater willingness to use social media (Alsuhaymi, 2018). Chuang (2020) confirmed the positive impact of social identity on user engagement behaviors in online communities. Prentice et al. (2019) discovered that individuals who engage in social activities form a feeling of belonging and social identity by interacting with their society, which supports this argument. Furthermore, when users receive invitations from people with similar social identities, they are more likely to remain active and engaged in the network for longer periods (Lento et al., 2006). On the other hand, religion is one of the most influential value systems and also serves as a guide for social behavior and interaction (Kashif et al., 2017; Parida and Gadekar, 2024), providing us with social identity (Ysseldyk et al., 2011). Greenfield and Marks (2007) suggested that religious social identity moderates more frequent attendance at religious rituals.

Moreover, according to Social Identity Theory, when a specific social identity is prominent, individuals are inclined to conform to the group’s values and ideas. They demonstrate a desire to enhance their affiliation with the group (Jiang et al., 2016; Rhoudri and Ougoujil, 2024; Rudra, 2024). Researchers have discovered that subjective norms and intentions are positively associated, but this relationship only holds true for those who have a strong sense of identification with the group (Fielding et al., 2008; Jiang et al., 2016; Milanov et al., 2014). A separate research study discovered that social identity completely mediates the impact of subjective norms on intentions (Kashif et al., 2017; Jiang et al., 2016). Therefore, this study further proposes that users’ social identity influences their intentions toward digital religious behavior, and users’ adjustments to their attitudes toward social media content, the expectations of their social groups regarding certain behaviors, and users’ perceived controllability of these behaviors are related to their identification with their social groups. Based on the above perspective, social identity can serve as a mediating variable between the three variables of attitudes toward digital religion, subjective norms, perceived behavioral control, and digital religious intentions.

#### 2.7.2 Empathetic willingness as a mediator

Empathetic willingness is an important component of this construct because it captures the motivation behind digital religious behavior. Empathy refers to the tendency of individuals to share and understand the emotional states of others during interactions (Schutte and Stilinović, 2017). Empathetic willingness indicates the extent to which young people are willing to engage in religious and spiritual comforting activities and how much they plan to participate in such behaviors (Machackova et al., 2016; Salehzadeh, 2024). Ziebertz (2018) noted a strong positive correlation between empathy and digital religious behavior.

Some studies on online communities provide empirical evidence on the impact of empathetic willingness on participation and contribution behaviors (Wu and Li, 2021). Trust, social identity, and empathetic willingness are positively correlated with the willingness of online community members to contribute knowledge and support (Zhao et al., 2013). Therefore, this study further proposes that users’ empathetic willingness may form a positive attitude toward digital religion by seeking others’ attention or emotional exchange with others. Additionally, users may strengthen the subjective norms supporting this activity on social media due to their willingness to respond empathetically to others.

Furthermore, empathetic willingness may strengthen users’ perceived control over supporting digital religious activities on social media, thereby increasing their willingness to engage in such behaviors (Taravian and Nikdel, 2024). Chen and Ma (2023) pointed out that the sources influencing empathy are the social media production itself, with interesting, vivid, unique, and emotional topics being more likely to stimulate users’ empathetic willingness and promote everyday behavior formation. Chen and Ma (2023) further confirmed that empathetic willingness has a positive impact on social media users’ participation in online behaviors. Rawal and Saavedra Torres (2017) noted that empathetic willingness plays a mediating role between social network users’ participation attitudes and participation behaviors. Therefore, this study also examines the mediating role of empathetic willingness.

### 2.8 Research framework and hypotheses

According to the researchers’ understanding, in the few empirical studies investigating religious behavior among Chinese youth, the assessment of religious behavior usually focuses only on offline religious activities (Feuchtwang, 2003; Chau, 2008; Lin, 2020) or online religious activities (Shen, 2023; Xia, 2023), with few studies including both or more common forms of religious behavior. Additionally, previous research has identified that the type of assessment used plays an important role in determining attitudes and the frequency of religious behavior (Campbell, 2017). Therefore, the type of evaluation can impact the way respondents perceive and their readiness to disclose religious behaviors. This study encompasses three categories of activities: online prayer, offline religious behaviors prompted by online content, and concurrent online and offline religious behaviors. These three are the most common means of assessing digital religious behavior among Chinese youth.

This study employs the TPB model to explain digital religious behavior among Chinese youth. Furthermore, this study aims to specifically investigate the direct impact of social identity and empathetic willingness, as well as their intermediary effects on digital religious behavior. We have formulated the following hypotheses based on the discussion above.

H1: There is a positive correlation between DRI and DTB among Chinese young social media users.

H2: SN (H2a), ATDR (H2b), and PBC (H2c) positively predict DRI among Chinese young social media users.

H3: SI (H3a) and EWs (H3b) will have a positive direct impact on DRI among Chinese young social media users.

H4: EW mediates the relationship between SN (H4a), ATDR (H4b), PBC (H4c), and DRI.

H5: SI mediates the relationships between SN (H5a), ATDR (H5b), PBC (H5c), and iDRI.

H6: SN (H6a), ATDR (H6b), and PBC (H6c) positively influence SI, which in turn positively influence DRI and subsequently DRB.

H7: SN (H7a), attitudes (H7b), and PBC (H7c) positively influence EW, which in turn positively influences DRI and subsequently DRB.

H8: EW affects DRI by enhancing the degree of influence of SI.

## 3 Method

### 3.1 Sample

The study distributed surveys to 30,000 eligible online users via the “@” feature on Sina Weibo and the private messaging function on Douyin (10,000 Weibo users and 20,000 Douyin users). Ultimately, 541 respondents provided valid responses and received a reward of 30 RMB (approximately 4.80 USD). After data cleaning, the final number of valid questionnaires was 525 (236 male, 289 female; response rate: 97.03%). In China, Sina Weibo and Douyin, two of the most popular social media platforms among Generation Z (Chen and Yang, 2023). It is evident that a significant proportion of Generation Z in China actively uses these platforms, making them suitable for our research (e.g., Comendulli, 2020; Verot, 2023; Zhang, 2024). While it is true that not all members of Generation Z are active on these platforms, the vast majority are. Our sampling method ensures that we capture a broad and diverse segment of this population, reflecting various demographic factors such as age, gender, and geographic location. This diversity within the user base helps mitigate potential biases and enhances the representativeness of our sample. Moreover, this research aims to understand the nuances of social media behavior among Generation Z. By targeting platforms where this demographic is most active, we ensure that our findings are directly relevant and applicable.

### 3.2 Measurement

Demographic information, including age, gender, and educational level, was collected from participants.

Measurement items were adapted from published literature and TPB questionnaires available on the TPB website (Ajzen, 2015), to better suit the social digital religious behaviors of Chinese youth. All items used a 5-point Likert scale. The brief description of the scales is as follows:

- *Digital Religious Intention (DRI)*: Assessed using 5 items (e.g., “I intend to participate in ‘seeking deities and offering prayers’ activities through social media”), rated from 1 (very unlikely) to 5 (very likely), to evaluate respondents’ intentions toward digital religious activities.
- *Attitudes Toward Digital Religion (ATDR)*: Evaluated using 6 items (e.g., “The method of ‘seeking deities and offering prayers’ on social media is useful to me”), rated from 1 (strongly disagree) to 5 (strongly agree), to gauge respondents’ attitudes towards digital religion.
- *Subjective Norms (SN)*: Measured using 6 items (e.g., “The frequency with which my family or friends recommend ‘seeking deities and offering prayers’ activities”), rated from 1 (never) to 5 (always), to assess the influence of recommendations from close relations regarding various forms of digital religious behaviors.
- *Perceived Behavioral Control (PBC)*: Measured using 5 items (e.g., “Seeking deities and offering prayers on social media is easy for me”) to measure the likelihood of engaging in digital religious activities.
- *Digital Religious Behavior (DRB)*: Assessed using 5 items (e.g., “The frequency of participating in online ‘seeking deities and offering prayers’ activities through social media”), rated from 1 (never) to 5 (always), to evaluate the frequency of various forms of digital religious behavior reported by respondents.
- *Social Identity (SI)*: Measured using 5 items (e.g., “Participating in ‘seeking deities and offering prayers’ on social media makes me feel a sense of belonging”), rated from 1 (strongly disagree) to 5 (strongly agree).
- *Empathetic Willingness (EW)*: Measured using 5 items (e.g., “Participating in ‘seeking deities and offering prayers’ activities helps me emotionally relax when I am upset/frustrated/stressed”), rated from 1 (strongly disagree) to 5 (strongly agree).

Every question in the questionnaire was specifically created to aid students who have a lesser level of ability in the English language. To confirm the accuracy of the translation, a conventional back-translation technique was utilized (Brislin, 1986). A pilot study was conducted with a sample of 31 students to verify the clarity of the questionnaire items and to identify any potential difficulties (Lin, 2020). Additionally, three experts, each boasting over a decade of experience in media and cultural studies, provided validation for the questionnaire. We developed the final version of the instrument based on feedback from these experts and recommendations from the pilot study. The questionnaire was deemed ready for use in a real study involving Generation Z youth in China. As shown in Table 1, the Cronbach’s alpha coefficients for all variables exceed 0.9, indicating excellent internal consistency and high reliability of the scale.

**Table 1:** Reliability.

Constructs	Cronbach’*α*
Social norms	0.931
Attitudes towards digital religion	0.936
Perceived behavioral control	0.931
Social identity	0.900
Empathetic willingness	0.925
Digital religious intention	0.944
Digital religious behavior	0.955

## 4 Results

### 4.1 Demographic results

The demographic characteristics of the participants are presented in Table 2. In the sample, about 44.95% of the participants were males, and the others were females. A majority of participants had undergraduate education, with 70%, and a share of 29.14% had postgraduate education. All participants reported no religious affiliation. This distribution reflects a highly educated sample, with 70.8% of users holding an associate degree or higher (Sina Weibo Data Center, 2013). On Douyin, the age group of 18–25 constitutes the largest segment, comprising 23.5% of all users (QuestMobile, 2023).

**Table 2:** Demographics of respondents (*N* = 525).

	M	Percent
Gender		
Male	236	44.95
Female	289	55.05
Age		
18-19	179	34.09
20–22	193	36.76
22–25	153	29.14
Educational background		
Undergraduate	372	70.85
Postgraduate	153	29.14
Religion		
Christianity	0	0
Buddhism	0	0
Others	0	0

### 4.2 Confirmatory factor analysis (CFA) model

Collier (2020) states that Structural Equation Modeling (SEM) analysis begins with Confirmatory Factor Analysis (CFA), a theory-based method used to verify the measurement model (Palazzo and Foroudi, 2024) of underlying constructs. Consequently, Confirmatory Factor Analysis (CFA) serves as an initial application of validation to evaluate the measurement models. This provides a highly efficient technique for analyzing and validating study conceptions in terms of accuracy and consistency. Table 3 presents the standardized factor loadings (SFL), composite reliability (CR), and average variance extracted (AVE) for each construct, as derived from the CFA analysis.

**Table 3:** SFL, CR, AVE of items of the academic dishonesty questionnaire.

Constructs	SFL	CR	AVE
Social norms (Griffin et al., 1987)			
The frequency with which my family recommends “seeking deities and offering prayers” influences me.	0.866	0.931	0.692
The frequency with which my friends recommend “seeking deities and offering prayers” influences me.	0.762		
The frequency with which some groups I belong to share “seeking deities and offering prayers” on social media influences me.	0.817		
My family often sharing “seeking deities and offering prayers” content on social media influences me.	0.862		
My friends often sharing “seeking deities and offering prayers” content on social media influences me.	0.813		
Some groups I belong to sharing “seeking deities and offering prayers” content on social media influences me.	0.867		
Attitudes towards digital religion (Molinillo et al., 2018)			
The method of “seeking deities and offering prayers” on social media is useful to me.	0.839	0.938	0.716
The method of “seeking deities and offering prayers” on social media is convenient for me.	0.84		
The content of “seeking deities and offering prayers” shared on social media is helpful to me.	0.864		
The content of “seeking deities and offering prayers” shared on social media is helpful to me.	0.854		
Any “seeking deities and offering prayers” activity should be taken seriously.	0.826		
“Seeking deities and offering prayers” can help society relieve stress.	0.854		
Perceived behavioral control (Wang and Wong, 2021)			
“Seeking deities and offering prayers” on social media is easy for me.	0.892	0.933	0.736
Offline “seeking deities and offering prayers” is easy for me.	0.884		
Accessing “seeking deities and offering prayers” content on social media is easy for me.	0.892		
Whether I engage in “seeking deities and offering prayers” is entirely up to me.	0.845		
I have the resources, time, and opportunities to participate in online/offline “seeking deities and offering prayers.”	0.77		
Social Identity (Jiang et al., 2016)			
I enjoy participating in “seeking deities and offering prayers” on social media with friends.	0.815	0.901	0.646
Participating in “seeking deities and offering prayers” on social media makes me feel a sense of belonging.	0.839		
I like being a part of the youth “seeking deities and offering prayers” community.	0.811		
I identify with other members of the youth “seeking deities and offering prayers” community.	0.822		
The youth “seeking deities and offering prayers” community is an important reflection of who I am.	0.728		
Empathetic willingness (Alhabash et al., 2013)			
Participating in “seeking deities and offering prayers” activities helps me emotionally relax when I am upset/frustrated/stressed.	0.888	0.926	0.714
I feel happy when participating in “seeking deities and offering prayers” activities.	0.855		
Sharing or interacting with others about “seeking deities and offering prayers” on social media satisfies my emotional needs.	0.793		
Participating in “seeking deities and offering prayers” activities positively impacts my sense of empathy.	0.834		
I feel happy when I know that my friends or acquaintances are participating in “seeking deities and offering prayers” activities on social media.	0.851		
Digital religious intention (Hwang, 2018)			
I consider participating in “seeking deities and offering prayers” activities through social media.	0.46	0.902	0.657
I consider attending offline “seeking deities and offering prayers” activities when informed through social media.	0.883		
I consider sharing “seeking deities and offering prayers” activities on social media with others.	0.856		
I consider recommending others to participate in offline “seeking deities and offering prayers” activities.	0.869		
I would participate in “seeking deities and offering prayers” activities if given the opportunity.	0.898		
Digital religious behavior (Vilchinsky and Kravetz, 2005)			
The frequency of participating in online “seeking deities and offering prayers” activities through social media.	0.887	0.955	0.811
The frequency of participating in offline “seeking deities and offering prayers” activities when informed through social media.	0.9		
The frequency of sharing “seeking deities and offering prayers” activities through social media.	0.922		
The frequency of bringing others to participate in online “seeking deities and offering prayers” activities.	0.899		
The frequency of bringing others to participate in offline “seeking deities and offering prayers” activities.	0.894		

SFL, standardized factor loadings; CR, composite reliability; AVE, average variance extracted.

The correlation coefficients of the questionnaire items are presented in Table 4. The CFA measurement model for the seven constructs shows that all items have standardized factor loadings (SFLs) larger than 0.40. Stevens (2002) suggests that SFLs larger than 0.40 are usually thought to be significant, irrespective of sample size. The composite reliability (CR) ranges from 0.763 to 0.922, all beyond the acceptable criterion of 0.70 (Sarstedt et al., 2021), suggesting strong internal consistency and convergent validity. The AVE values vary from 0.646 to 0.811, which exceeds the minimum value of 0.5 suggested by Fornell and Larcker (1981), hence confirming convergent validity (Bhutto et al., 2024). This finding offers compelling support for the concept of discriminant validity. The CFA analysis has determined that the obtained data and the proposed CFA measurement model are considered completely appropriate (Cohen et al., 2013). In order to confirm the validity of the research hypotheses in this study, the next step is to evaluate the fit indices of the suggested structural model (Rex, 2023). Furthermore, the square root values of AVE for each latent variable on the diagonal exceed the correlation coefficients between the variable and other latent variables, suggesting strong discriminant validity among the constructs in the model (Xiao and Ke, 2024).

**Table 4:** Correlations between variables.

	1	2	3	4	5	6	7
1. SN	**0.832**						
2. ATDR	0.472***	**0.846**					
3. PBC	0.523***	0.571***	**0.858**				
4. SI	0.540***	0.650***	0.638***	**0.804**			
5. EW	0.475***	0.570***	0.577***	0.635***	**0.845**		
6. DRI	0.359***	0.464***	0.470***	0.534***	0.471***	**0.811**	
7. DRB	0.319***	0.335***	0.404***	0.386***	0.399***	0.482***	0.9

Squared root of AVE values are noted on the diagonal with bold.

*Correlation is significant at 0.05 level. **Correlation is significant at the 0.01 level. ***Correlation is significant at the 0.001 level (two-tailed).

### 4.3 Structural model analysis

An assessment of the structural model is necessary to determine the adequacy of the model fit and ensure that all three categories of model fit indices are met (Juan et al., 2022). The relevant fit indices include: (1) Absolute Fit: Chi-square and Root Mean Square Error of Approximation (RMSEA); (2) Comparative Fit Index (CFI); (3) Parsimonious Fit: Chi-Square/df. The model testing validated the model that elucidates digital religious behavior among Chinese youth. The statistical measures of the model’s fit are displayed in Table 5. According to the model fit indices, generally, the chi-square to degrees of freedom ratio (*χ*^2^/df) should range between 0 and 5, with a smaller *χ*^2^/df value indicating a stronger model fit. In this model, the *χ*^2^/df value is 2.265, which falls within the acceptable fit reference range. The Goodness of Fit Index (GFI), Comparative Fit Index (CFI), Tucker-Lewis Index (TLI), and Normed Fit Index (NFI) are indices with a value range between 0.85 and 1. A value closer to 1 represents a higher degree of fit. In this model, the GFI is calculated to be 0.880, the CFI is 0.955, the NFI is 0.922, and the TLI is 0.951. All four indices exceed the 0.85 threshold, indicating a satisfactory fit. The Root Mean Square Error of Approximation (RMSEA) is an index that evaluates the extent of model misfit, with a range between 0 and 0.08. In this study, the RMSEA is calculated to be 0.049, which is within the acceptable fit reference range. In summary, all the measurement indices are within the fit standards, indicating that the model has a satisfactory fit with the sample data.

**Table 5:** Fit statistics for model.

Fit index	Satisfied	Model	Evaluation rationale
*χ* ^2^	N/A	1386.001	N/A
df	N/A	612	N/A
RMSEA	0 < RMSEA <0.08	0.049	West et al. (2012)
GFI	0.85 < GFI < 1.00	0.880	Loehlin (2004)
CFI	0.85 < GFI < 1.00	0.955	Schermelleh-Engel et al. (2003)
NFI	0.85 < GFI < 1.00	0.922	Kline (2023)
TLI	0.85 < GFI < 1.00	0.951	Leguina (2015)
*χ*^2^/df	0 < *χ*^2^/df < 5	2.265	McDonald and Ho (2002)

### 4.4 Testing the hypotheses

Building on the validation of measurement results using existing datasets, the next step in this study is to test the hypotheses using Structural Equation Modeling (SEM). To test the research hypotheses, we utilized IBM-SPSS-AMOS 24.0 software and employed unstandardized estimates and regression weights to evaluate the proposed relationships.

**Figure 1:**
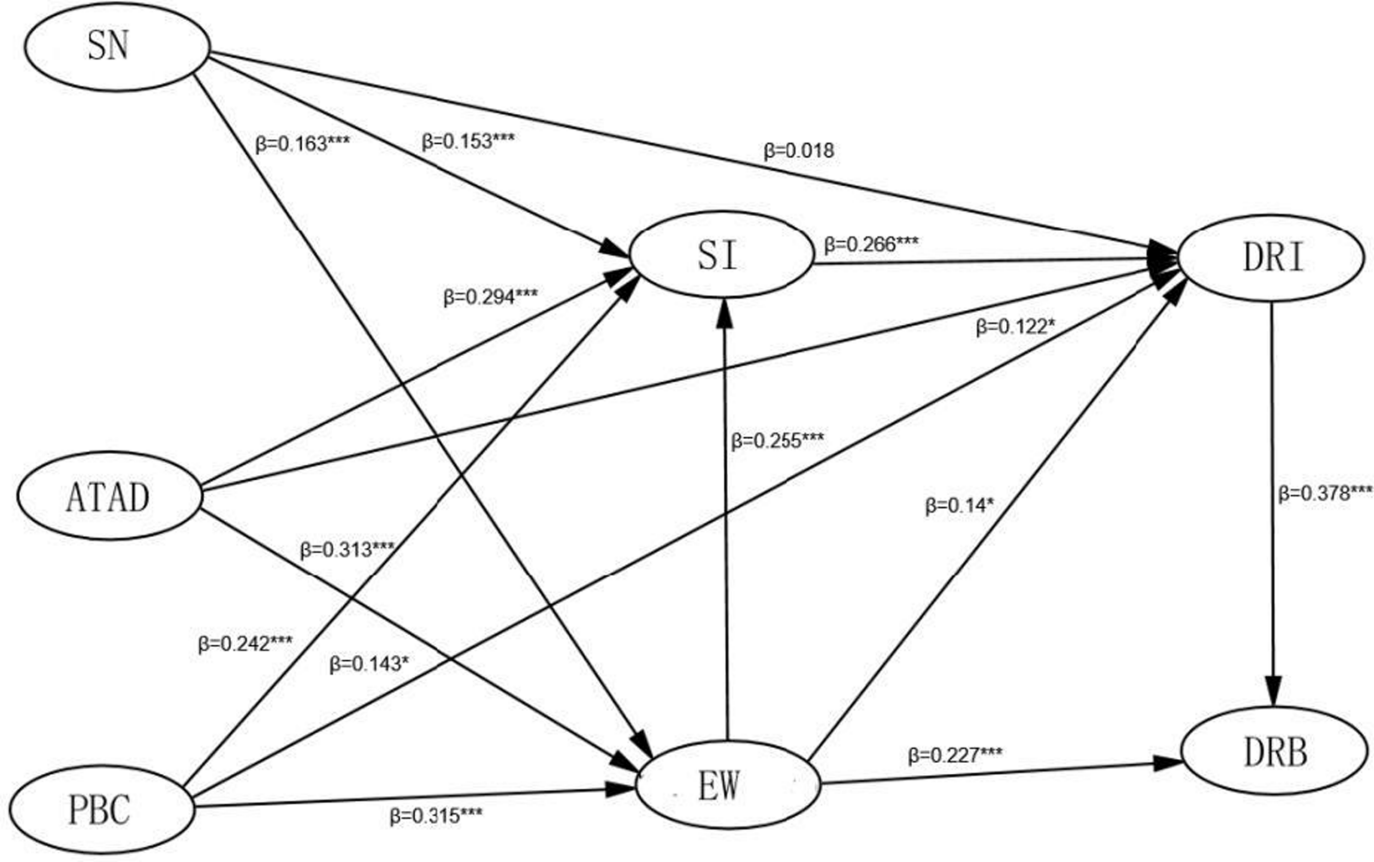
Results of structural equation model. *p < 0.05; **p < 0.01; ***p < 0.001.

Table 6 presents the standardized regression coefficients from the path analysis, which aims to test the significance of the relationships between variables and assess the support for the hypotheses based on the collected data. Based on the SEM, Figure 1 illustrates the standardized estimates proposed in the structural model.

**Table 6:** Standardized path coefficients to testing the causal effects of the constructs for model.

Hypothesis	Construct	Path	Construct	Estimate	S.E.	C.R.	*p*	Result
H1	DRI	→	DRB	0.378	0.064	6.611	***	Support
H2a	SN	→	DRI	0.018	0.058	0.361	0.718	Reject
H2b	ATDR	→	DRI	0.122	0.05	2.107	0.035	Support
H2c	PBC	→	DRI	0.143	0.061	2.427	0.015	Support
H3a	SI	→	DRI	0.266	0.08	3.736	***	Support
H3b	EW	→	DRI	0.14	0.05	2.409	0.016	Support

***p < 0.001.

As shown in Table 6, Social Identity (SI) (*β* = 0.266, *p* < 0.001) has the strongest direct predictive effect on intention, followed by Perceived Behavioral Control (PBC) (β = 0.143, *p* < 0.05) and Empathetic Willingness (EW) (β = 0.14, *p* < 0.05), thereby supporting hypotheses H2b, H2c, H3a, and H3b. However, Subjective Norms (SN) (β = 0.018, *p* > 0.05) did not have a significant direct effect on intention, leading to the rejection of hypothesis H2a. Additionally, SN (β = 0.294, *p* < 0.001), PBC (β = 0.242, *p* < 0.001), and Attitudes Toward Digital Religion (ATDR) (β = 0.294, *p* < 0.001) were positively correlated with SI (β = 0.32, *p* < 0.01) and significantly related to EW. SI significantly influenced ATDR, fully supporting hypothesis H4b. EW also positively impacted SI (β = 0.62, *p* < 0.01). Finally, Digital Religious Intention (DRI) significantly predicted Digital Religious Behavior (DRB) (β = 0.378, *p* < 0.001), fully supporting hypothesis H1.

### 4.5 Mediating effect

To further investigate the mechanisms through which Social Identity (SI) and Empathetic Willingness (EW) influence Digital Religious Behavior (DRB), we examined the indirect effects mediated by these variables. Table 7 provides a summary of the data results. The significance of the mediating effects was assessed using the ratio of the indirect effect to the standard error (SE) being 1.96 or the confidence interval of the percentile method and bias-corrected procedure containing a non-zero value (Ledermann and Macho, 2009). Generally, partial mediation occurs when the direct effect remains significant, while full mediation occurs when the direct effect is no longer significant (Morgan, 2013). This study reveals significant mediating effects of Subjective Norms (SN), Attitudes Toward Digital Religion (ATDR), and Perceived Behavioral Control (PBC) on Digital Religious Intention (DRI) through the variables of SI and EW.

**Table 7:** Test for mediating effects.

Hypothesis	Path	Estimate	Bootstrap 95%CI		*p*	Results
			Lower	Upper		
H4a	SN → SI → DRI	0.047	0.021	0.088	0	Support
H4b	ATDR→SI → DRI	0.067	0.039	0.108	0	Support
H4c	PBC → SI → DRI	0.066	0.038	0.113	0	Support
H5a	SN → EW → DRI	0.027	0.008	0.054	0.009	Support
H5b	ATDR→EW → DRI	0.038	0.014	0.067	0.011	Support
H5c	PBC → EW → DRI	0.046	0.018	0.083	0.009	Support
H6a	SN → SI → DRI → DRB	0.02	0.009	0.04	0	Support
H6b	ATDR→SI → DRI → DRB	0.028	0.016	0.05	0	Support
H6c	PBC → SI → DRI → DRB	0.028	0.016	0.053	0	Support
H7a	SN → EW → DRI → DRB	0.011	0.004	0.024	0.006	Support
H7b	ATDR→EW → DRI → DRB	0.016	0.006	0.029	0.01	Support
H7c	PBC → EW → DRI → DRB	0.019	0.008	0.037	0.006	Support
H8	EW → SI → DRI	0.058	0.033	0.102	0	Support

Specifically, the mediation effect of SN → SI → DRI is 0.047, with a 95% confidence interval of (0.021, 0.088), excluding zero, indicating a significant mediation effect. The mediation effect of ATDR→SI → DRI is 0.067, with a 95% confidence interval of (0.039, 0.108), excluding zero, indicating a significant mediation effect. The mediation effect of PBC → SI → DRI is 0.066, with a 95% confidence interval of (0.038, 0.113), excluding zero, indicating a significant mediation effect, thus fully supporting H4 and H5. Additionally, the mediation effect of SN → EW → DRI is 0.027, with a 95% confidence interval of (0.008, 0.054), excluding zero, indicating a significant mediation effect. The mediation effect of ATDR→EW → DRI is 0.038, with a 95% confidence interval of (0.014, 0.067), excluding zero, indicating a significant mediation effect. The mediation effect of PBC → EW → DRI is 0.046, with a 95% confidence interval of (0.018, 0.086), excluding zero, indicating a significant mediation effect, thus fully supporting H4.

Moreover, the data indicate that the chain multiple mediation of SN → SI → DRI → DRB is 0.002, with a 95% confidence interval of (0.009, 0.004), excluding zero, indicating the presence of a chain multiple mediation effect. The chain multiple mediation of ATDR→SI → DRI → DRB is 0.028, with a 95% confidence interval of (0.016, 0.005), excluding zero, indicating the presence of a chain multiple mediation effect. The chain multiple mediation of PBC → SI → DRI → DRB is 0.028, with a 95% confidence interval of (0.016, 0.053), excluding zero, indicating the presence of a chain multiple mediation effect. The chain multiple mediation of SN → EW → DRI → DRB is 0.011, with a 95% confidence interval of (0.004, 0.024), excluding zero, indicating the presence of a chain multiple mediation effect. The chain multiple mediation of ATDR→EW → DRI → DRB is 0.016, with a 95% confidence interval of (0.006, 0.029), excluding zero, indicating the presence of a chain multiple mediation effect. The chain multiple mediation of PBC → EW → DRI → DRB is 0.019, with a 95% confidence interval of (0.008, 0.037), excluding zero, indicating the presence of a chain multiple mediation effect. Therefore, H6 and H7 are accepted.

## 5 Limitations

We identified several limitations that warrant caution in interpreting these results. Firstly, the results discussed may not reflect the digital religious behavior of all Generation Z youth in China. Secondly, self-reported measures might be subject to social desirability bias, potentially leading to underreporting of digital religious behavior (Whitley, 1998). Thirdly, the dimensions of digital religious behaviors in the study are not strictly delineated into online or offline categories, as this distinction is not the focus of our research. Therefore, a limitation of our work is the lack of detailed examination of the relationship between specific digital religious behaviors and their influencing factors. Another limitation is the reliance on quantitative and cross-sectional research. Future studies should include qualitative components to explore youth digital religious behavior in depth. Additionally, the dimensions for measuring digital religious behavior should be more comprehensive, such as the presentation and attractiveness of digital religious audiovisual content, which are loosely connected to the fundamental causes of digital religious behavior. Future research should focus more on the psychological mechanisms of youths’ digital religious behavior. Researchers call for continued study and learning in this area, as it remains a focal point of media studies and youth research.

## 6 Discussion

This study uses an extended TPB to examine the psychological determinants of digital religious intentions and digital religious behaviors among Chinese youths. The results of the path analysis provide fundamental support for the validity of the extended TPB in interpreting the willingness of Chinese youths to engage in digital religion via social media platforms. As expected, intention significantly predicts digital religious behavior among Chinese youths. Attitude towards digital religion, social identity, empathetic willingness, and perceived behavioral control have significant direct effects on digital religious intention. Significantly, this study discovers evidence for the first time that empathetic willingness has a statistically significant impact on digital religious intentions.

PBC is significantly related to the tendency for digital religious behavior. This finding is consistent with previous research (Fu and Cook, 2020; Gangneux, 2019). Social media provides young people with personal empowerment, broader access and connectivity, means for community and identity formation, tools to organize their social lives, and various interaction possibilities that might be limited by adult mediation and physical space (Stahl and Literat, 2023; Zeng and Abidin, 2023). Furthermore, Chinese youth on social media will have a strong sense of behavioral control and a high willingness to participate in digital religion when they realize that they can easily access the opportunities and resources needed to succeed in performing religious acts.

Additionally, empathetic willingness is the strongest predictor of digital religious behavior, consistent with previous studies (Ziebertz, 2018; Machackova and Pfetsch, 2016). In contemporary China, young people face tremendous pressure from workplace competition, social interactions, and economic stress, leading to feelings of anxiety, confusion, indecision, and fear of making the wrong choices. Thus, on one hand, they seek online religious avenues to gain understanding from others through shared experiences, achieving emotional relief and comfort. On the other hand, they hope to use spiritual beliefs to relieve anxiety, resolve confusion, and gain psychological comfort through emotional exchanges (Xia, 2023; Zhang, 2023; Wu, 2008). Therefore, online religion can be seen, to some extent, as a form of social technology empowerment, offering contemporary youth a new “pressure valve” for emotional contribution, comfort, and tension reduction.

In the model, subjective norms are not predictors of intention but are significantly related to social identity and empathetic willingness. This finding is inconsistent with previous research (Jiang et al., 2016; Idris et al., 2016). A society with strong atheistic tendencies can explain why the perceived views of social group members (subjective norms) are not determinants of digital religious behavior intentions (Xia, 2023). Unlike the social contexts of theistic countries (e.g., North America or European countries with Christian beliefs, or Muslim countries), digital religious behavior among contemporary Chinese youth is a temporary, non-religious activity, undertaken only as a comforting behavior when anxiety cannot be resolved or exceeds the tolerance limit. The Chinese government has advocated atheism since the founding of the People’s Republic in 1949 (Ashiwa and Wank, 2009). Under the Communist Party’s philosophy, atheism was established as the state-sanctioned perspective, and formal religious rituals were actively suppressed, especially among older generations (Ter Haar, 2021). This has resulted in an environment where atheism and secularism are integrated into educational systems, public institutions, and media. Young individuals are frequently reared with limited exposure to formal religious rituals. China’s educational framework prioritizes scientific and logical thought, corresponding with secular and atheistic philosophies (Han and Ye, 2017). The curriculum typically excludes religious education, resulting in a culture where religion is not a prominent aspect of the mainstream perspective. This has led younger generations to perceive religion as a personal matter rather than a public concern, frequently linking it to antiquated traditions or foreign cultures.

For numerous Chinese youths, religious identification is subordinate to national, cultural, or familial identity. This has fostered a developing identity among youth that often emphasizes cultural heritage and social ideals over religious allegiance. Atheism frequently positions religion as a subject of historical or cultural inquiry rather than a matter of spiritual devotion. Notwithstanding secular influences, numerous young individuals are progressively intrigued by the examination of classic Chinese philosophies and cultural practices, including Confucianism, Taoism, and Buddhism (Chen and Ma, 2023). These traditions are frequently seen not as religious rituals but as sources of ethical instruction, cultural pride, and personal growth. Confucian ideas, which prioritize reverence for elders, familial loyalty, and societal harmony, are regarded as fundamental components of social and familial identity (Wu and Sun, 2024). Chinese adolescents frequently engage in spiritual practices in a secular manner, emphasizing meditation, mindfulness, or traditional festivals that resonate with cultural identity rather than adhering to official religious affiliations. This illustrates a wider trend of syncretism, where youth may amalgamate aspects of other philosophies or religions based on personal significance rather than doctrinal allegiance. Chinese youth typically regard organized religion with skepticism, frequently viewing it as incongruent with contemporary principles or personal autonomy. This perspective is somewhat influenced by historical instances of religious constraints, which cultivated skepticism toward organizations perceived as repressive or antiquated. A growing number of young individuals are becoming connected to global ideas via social media and international schooling, thereby encountering a variety of religious and spiritual viewpoints. Some individuals embrace or investigate religious beliefs as components of a global cultural identity; however, they generally endeavor to amalgamate these with their Chinese identity.

Therefore, according to Campbell’s approach, we suggest not viewing digital religion as a fixed entity; under the new conditions created by media technology for religious practice, religious practice is becoming increasingly “flexible.” Rethinking the terms used to describe digital religion can also help explain contemporary high-tech societies where much interpersonal communication, including religious experiences, occurs through digital media (Giulia, 2022). Furthermore, this indicates that the reasons for the digital religious behavior of contemporary Chinese youth stem from the pressures caused by the changing times and social environment, and represent their efforts at self-reflection, self-breakthrough, and self-healing when facing difficulties. Additionally, the significant correlation between social identity and empathetic willingness suggests that structural social factors are powerful external forces shaping individual religious characteristics (Campbell and Evolvi, 2020). Humans are not passive, unquestioningly shaped by society; individuals are active agents who creatively and adaptively modify their roles.

The results also indicate that attitude is significantly related to the intention and behavior of digital religion, consistent with previous studies (e.g., Luchsinger, 2020; McKeever, 2015; Sisler, 2017). From a structural perspective, this reflects the structural issues specific to certain groups in China, closely related to the overall structure of Chinese social development. It mirrors the psychological dynamics, behavior patterns, and cultural concepts of the youth group, blending multiple psychological states such as anxiety, aspiration, confusion, and hesitation among Chinese youth. It also points to the real-life dilemmas and survival situations of contemporary youth during times of societal transformation. Digital technology breaks the materialized space for traditional religious communities to mediate personal spiritual activities, making “internet spiritualization” a framework suitable for religious experiences (Campbell and Evolvi, 2020), i.e., creating “forms of feeling” through media narratives that negotiate meaning, spiritual interaction, and religion (Birgit, 2010). Attitudes and practices help construct transcendent experiences that “spiritualize the internet.”

Furthermore, the study shows that social identity fully mediates the relationship between subjective norms, attitude, perceived behavioral control, and digital religious intention. The results highlight the critical role of social identity in the practice of digital religious behavior among young social media users. The results indicate that social intention is the primary factor driving digital religion intention, suggesting that Chinese youth interact on social media for the purpose of forming a sense of group identity.

The Pew Research Center (2021) suggests that Generation Z easily shares their voices in their own ways in cyberspace. Social media serves as the “home ground” for Generation Z (Maguire, 2021; Stahl and Literat, 2023) and a crucial space for youth’s online collective expression (Literat and Kligler-Vilenchik, 2019). It is a valuable window to understand youth attitudes and views, including their expression of generational identity. As Kong (2010) writes, technological development opens new spaces for religious practice, identity formation, and community formation. The changing social and spatial strategies of different religious communities contribute to creating what scholars call a post-secular society or “super-diverse” society (Evolvi, 2022; Becci et al., 2017). Media researchers discuss potential online discourse performances through spatial metaphors (Nelson et al., 2020) or proximate spatiality (Campbell, 2017). In this context, Chinese youth transform the networked practices of social media into a “third space” of digital religion (Yamane, 2016), facilitating the negotiation of hybrid identities and aesthetic imaginations mediated by platforms.

On the other hand, social identity theory posits that the process of socialization during personal growth involves distinguishing between self and others, aiming to achieve a positive social identity. Stahl and Literat (2023) position youth as a normative period for consolidating a sense of identity, where changes in identity in the early twenties are better conceptualized as a continuous or even enhanced development related to open choices and the expression of personal agency (Hadad and Schachter, 2011; Sun and Zhang, 2021; Ateş and Kölemen, 2024). Youth immersed in subcultures, lacking mainstream discourse power, find in social and participatory media platforms a vital and dynamic space to express their personal and social identities (Deogracias, 2015; Zeng and Abidin, 2023; Stahl and Literat, 2023). Social identity, with its cognitive and emotional evaluation elements, is activated in the context of digital religion (Cohen and Soukup, 2023). Digital religion in social media constructs a meaningful space for young users to share emotional experiences, express psychological confusion, and seek social support. This dynamic narrative process can accomplish individual self-construction through obtaining social identity. Therefore, it follows that the strong influence of social identity on digital religious intention and conduct is not surprising. The study focuses on youth aged 18–25, a period experiencing the necessary process of socialization, distinguishing self and others, and seeking positive social identity. Additionally, social identity is not a binary phenomenon, but the degree of social recognition for each category varies. Many identities and roles are not positively viewed, leading individuals to hide certain attributes in a diverse set of roles. The varied characteristics of individuals grant them more self-concept and possibilities. However, in the context of universal values and a busy real life, many attributes are submerged or even forgotten. Anonymity on social media provides these individuals with a channel to express themselves and find their groups. Religious behavior offers them special value reflection and emotional comfort in the safest manner.

The findings of this study underscore the pivotal role of empathetic willingness in the practices of digital religious behavior (DRB) among young social media users. Users of social media platforms tend to favor the expression of self-emotions and the sharing and interaction with members of established virtual communities, thereby generating discussion fervor within these groups and driving changes in their own behavior. Empathy, contrary to what some scholars have suggested, is not an overflow of emotions (Brown and Kuss, 2020) but is primarily influenced by cognitive coordination and emotional sharing, leading to a balanced psychological experience and overt behavioral intention (Chen and Ma, 2023). Thus, this study posits that young Chinese people use solemn and reverent religious practices on social media as emotional bonds, desiring to connect with others emotionally and thereby escaping from states of indifference or prolonged negative emotions. Moreover, EW fully mediates the relationship between social identity, digital religious intention, and digital religious behavior, consistent with previous findings (Westhuizen van der and Adelakun, 2023). Social activities mediated by social media become complex, involving not only self-other emotions but also interaction, cooperation, emotional sharing, joint action, and social identity capabilities. It can be said that the willingness and behavior of young social media users to practice digital religion are broadly subsumed under and interrelated with factors such as “empathy,” “shared emotions,” and “social identity,” consistent with prior research (e.g., Alhabash et al., 2013; Zhu et al., 2017). As scholars have pointed out, social media users utilize the contextual cues provided by the interaction itself (Butterfill, 2013) for a more complex experiential form. This complex form of experience transcends the ways in which traditional religious studies have shaped our understanding of media technology and the ways in which communication technology has complicated theology. It enters the realm of ideological and spiritual engagement bred by the experience of using media technologies—a form of “human intentionality and group agency” (Campbell and Evolvi, 2020), built upon more fundamental, more specific forms of interaction, particularly empathetic engagement.

Furthermore, EW reveals the internal motivation of the youth to seek spiritual solace and emotional support on social media. As an emerging form of spiritual practice in China, digital religious behavior precisely meets the emotional needs of the youth. This factor also indicates that the youth group, which has grown up under an atheistic backdrop, is forging new realms of spiritual practices. There is a strong link between social identity, empathetic willingness, and digital religious behavior, which suggests that structural social factors have a significant impact on how religious people are (Campbell and Evolvi, 2020). Humans are not passive recipients who accept social conditioning without question; rather, individuals are proactive agents who actively create and adaptively modify their roles. Further, the identification of these two key factors—social identity and empathetic willingness—provides important insights into the spiritual practices of Chinese youth in an atheistic context. Social media prompts individuals to construct their own online identities, negotiate and validate their identity claims (Khazraee and Novak, 2018; Xian, 2024), and most importantly, to obtain sufficient social recognition and support by building multiple identities (Stets and Serpe, 2016). Young people establish closer interpersonal relationships and strengthen their connections with others by sharing experiences of prayer and worship, life, and the exchange of gains and losses, thereby obtaining a sense of recognition and belonging.

Ultimately, digital religion and social media have added new dimensions to the spiritual practices of the Chinese youth group. Hypermediated spaces have created venues where people can post comments, craft narratives, and discuss their feelings. Social media, representing the third space, is an important site for religious experiences, providing a platform for the dissemination of information related to folk beliefs and personal views (e.g., Evolvi, 2022; Campbell and Evolvi, 2020). In essence, digital religious practices are religious narrative texts that are created through religious experiences, with a clear focus on social identification and empathy. In this process, the Chinese youth group has created a social space that exists between alternative and mainstream narratives, public and private emotions, and real and imagined religious experiences. Their online religious practices are not directed toward spiritual faith but rather serve as a means to counteract social alienation and seek emotional (or psychological) comfort. In the context where the Chinese government persists in advocating atheism and imposes restrictions and scrutiny on religious activities, contemporary Chinese youth have cultivated a self-centric spiritual practice through digital religious experiences (Wei, 2024). While experiencing the solace and attraction that religion provides to the soul and mind, individuals have rediscovered a sense of identity and emotional belonging through online religious communities.

## 7 Implications for theory and practice

Despite the fact that China’s Generation Z adolescents are highly engaged in digital religious behavior, there is a dearth of empirical research on this topic. This study provides a unique empirical investigation of religious practices and belief systems in China. We believe this is one of the largest investigations into the attitudes, norms, control, intentions, and behaviors regarding digital religion among Chinese youth. The findings provide valuable insights for Chinese scholars, educators, and overseas readers to gain a deeper understanding of the digital religious behavior, intentions, and motives of China’s Generation Z youth, both in the online and offline realms.

Social media serves as an effective vehicle for youth to articulate their views on God and disseminate their faith in meaningful, inclusive, and impactful manners via digital platforms. Young individuals may recount personal narratives illustrating how their faith has influenced their lives, molded their characters, or assisted them during difficult periods via social media platforms. Authenticity resonates on social media, and sharing genuine experiences can motivate others and illustrate the impact of faith on daily life. They may share creative artwork, photography, or digital designs that convey their convictions or illustrate topics of faith. Generation Z youth can articulate how their faith informs their perspectives on societal concerns such as mental health, poverty, equality, and justice, demonstrating that their faith encomp/asses not only worship but also the pursuit of a better world. Through these practices, social media can be used as an effective tool to express religious beliefs among Generation Z youth.

At the same time, funding from the government for youth programs that promote personal development through faith-based activities—such as mentorship, volunteering, or after-school initiatives—can assist young individuals in articulating their ideas in a positive manner. Educational institutions can establish programs that instruct students on global religions and cultural beliefs, promoting understanding, tolerance, and respect. This may diminish misconceptions and prepare students to participate in substantive debates. Religious communication may emphasize experiences and actions, such as community service initiatives, rather than solely theological instruction. Engaging youth in local service projects, mission endeavors, or worldwide humanitarian efforts enables them to actualize their convictions. With the use of social media platforms, effective policies, education, and religious communication efforts can be aligned to establish a supportive network that affirms young people’s needs for self-expression and personal growth through faith. A systematic strategy—where policies facilitate inclusive environments, education enhances comprehension, and religious dialogue actively involves and uplifts youth—can cultivate a generation that is both spiritually anchored and receptive to diverse perspectives.

Moreover, the theoretical contributions offer further validation for the use of an expanded model in forecasting and elucidating the religious behavior of adolescents in the digital realm. They also provide evidence for the efficacy of describing the decision-making processes of social media behavior, which are influenced by social identity and empathetic willingness, among Chinese youth. Specifically, it proposes for the first time that potential mediators, social identity, and empathetic willingness, are widely applied in the model of adolescent digital religious behavior. The TPB has been extensively utilized to examine the digital religious behavior of adolescents within the realm of social media, with the goal of fostering sustainable growth among this age group. This study could also be extended to other regions with unique cultural beliefs around the world, such as Taiwan and Japan, utilizing the research framework established here to investigate the relationship and influence between regional cultural practices and the digital religious behaviors of social media users in these areas.

## 8 Conclusion

This study is designed to comprehend the psychological elements and reasons that impact the social digital religious behavior of young Chinese individuals by utilizing data gathered from two social media platforms (Sina Weibo and Douyin). The fit indices indicate that the model proposed in this study is acceptable and has achieved a good fit, supported by the data collected from the investigated sample. The strong predictive power in this study once again demonstrates the robustness of the extended Theory of Planned Behavior model in the context of youth social media use behavior. Furthermore, the digital religious attitude and perceived behavioral control in the model significantly influenced the formation of digital religious intentions, while subjective norms were not a factor. This statistical result suggests that in the absence of religious beliefs in China, the reasons for the formation of intentions among youth are rooted in individual decision-making processes rather than the influence of close relationships or social norms.

The mediating effects of “social identity” and empathetic willingness indicate that the willingness and behavior of young social media users to practice digital religion are closely related to their needs for “empathy,” “shared emotions,” and “social identity.” This reflects the social mindset and living conditions of contemporary young people, characterized by anxiety, helplessness, confusion, and indecision, as well as a lack of available support in real life. Consequently, it is imperative to confront the desires and anticipations of the youth, provide guidance and assistance in fostering a constructive mindset, and prioritize cultivating empathy towards young individuals.

## 9 Future directions of research

This study has few limitations and provides future directions for the research. First, future research could explore developing a new model that places empathetic willingness as a core component rather than just a mediator. This could involve testing the direct effects of empathetic willingness on digital religious behavior and comparing the predictive power of this new model with the extended TPB. Our study is cross functional design, therefore, conduct longitudinal studies to examine how empathetic willingness and other predictors evolve over time and influence digital religious behavior. This can provide deeper insights into stability and changes in TPB relationships. Present study conducted in China culture and environment, therefore, we suggest extending the research to other cultural contexts to see if the findings hold true across different populations. This can help in understanding the universality or cultural specificity of the extended TPB model.

## Data Availability

The datasets presented in this study can be found in online repositories. The names of the repository/repositories and accession number(s) can be found in the article/Supplementary material.
